# Balancing mental health and work demands: Fitness-to-work assessment in a nurse with bipolar disorder

**DOI:** 10.1192/j.eurpsy.2026.11210

**Published:** 2026-07-31

**Authors:** C. Sridi, J. Ben Khelifa, N. Gannoun, N. Belhadj, F. Chelly, R. Nakhli, A. Fki, M. Maoua

**Affiliations:** 1 Occupational Medicine Department, Sahloul University Hospital; 2 University of Sousse, Faculty of Medicine of Sousse; 3 Research Laboratory LR19SP03; 4 Clinical Psychology Unit of Sahloul University Hospital, Sousse, Tunisia

## Abstract

**Introduction:**

The evaluation of fitness for work in healthcare professionals with psychiatric disorders presents complex challenges. Bipolar disorder, characterized by mood instability and functional impairment, raises specific concerns when considering high-stress and night-shift environments such as emergency departments.

**Objectives:**

To discuss the assessment of work fitness in a nurse with a history of bipolar disorder during a pre-employment medical evaluation, and to highlight the importance of tailored recommendations to ensure both patient safety and professional integration.

**Methods:**

We report the case of a 29-year-old nurse applying for a position in the emergency department of a University Hospital. During the occupational health examination, despite no initial medical history being reported, clinical observation revealed depressive affect. A systematic psychological evaluation in our service revealed a four-year history of bipolar disorder under mood stabilizer treatment, as confirmed by the treating psychiatrist.

**Results:**

Considering the risk factors associated with night shifts and stressful environments, the occupational physician issued restrictions, specifically the exclusion of night work and high-stress/conflictual contexts. Consequently, the assignment to the emergency department was not recommended. The nurse was redirected to an administrative position in a regional hospital with regular psychological follow-up.

**Conclusions:**

This case illustrates the crucial role of occupational health in balancing professional aspirations, workplace safety, and mental health considerations. Individualized work accommodations, based on psychiatric history and clinical evaluation, are essential for the sustainable employment of healthcare professionals with bipolar disorder.

**Disclosure of Interest:**

None Declared

